# A Novel Approach Based on Metabolomics Coupled With Intestinal Flora Analysis and Network Pharmacology to Explain the Mechanisms of Action of Bekhogainsam Decoction in the Improvement of Symptoms of Streptozotocin-Induced Diabetic Nephropathy in Mice

**DOI:** 10.3389/fphar.2020.00633

**Published:** 2020-05-21

**Authors:** Xianglong Meng, Junnan Ma, An Na Kang, Seok Yong Kang, Hyo Won Jung, Yong-Ki Park

**Affiliations:** ^1^Department of Herbology, College of Korean Medicine, Dongguk University, Gyeongju, South Korea; ^2^Experimental Teaching Center, College of Chinese Materia Medica and Food Engineering, Shanxi University of Chinese Medicine, Jinzhong, China; ^3^Korean Medicine R&D Center, Dongguk University, Gyeongju, South Korea

**Keywords:** Bekhogainsam decoction, diabetic nephropathy, metabolomics, gut microbiota, network pharmacology

## Abstract

Bekhogainsam decoction (BHID), a representative prescription for the treatment of diabetes mellitus (DM) and diabetic complications in both traditional Korean and Chinese medicine, was examined for its ability to ameliorate diabetic nephropathy (DN), and its mechanism of action was evaluated by metabolomics, gut microbiota, and network pharmacology. In this study, male specific pathogen-free C57BL/6 mice were intraperitoneally injected with streptozotocin (STZ, 100 mg/kg) once per day for 3 days consecutively, and were then orally administered BHID at 100 and 500 mg/kg, and metformin at 250 mg/kg once per day for 4 weeks. Our results showed that the administration of BHID to mice with STZ-induced DN prevented physiological and serological changes, structural damage, and kidney dysfunction. Based on a metabolomics test with serum, the profoundly altered metabolites in the BHID treatment group were identified. Thirty-six BHID-related proteins and four signaling pathways, including valine, leucine, and isoleucine biosynthesis, nicotinate and nicotinamide metabolism, tryptophan metabolism, and alanine, aspartate, and glutamate metabolism pathways, were explored. Principal coordinates analysis (PCoA) of the gut microbiota revealed that BHID treatment significantly affected the flora composition. In addition, the network pharmacology analysis revealed that BHID acted through phosphatidylinositol-3-kinase/protein kinase B (PI3K/Akt) and MAPK-related protein targets. Our findings on the anti-DN effects of BHID and its mechanism of action, from the perspective of systems biology, have provided scientific evidence to support the clinical treatment of patients with diabetes, and implied that BHID has the potential to prevent the progression of DN.

## Introduction

Diabetic nephropathy (DN), which is recognized as one of the most serious complications of type 2 diabetes mellitus (T2DM), is associated with proteinuria, hypertension, edema, and renal dysfunction and failure (Alicic et al., 2017). DN more commonly occurs in patients who have had T2DM for more than 10 years. However, its pathophysiology has not been fully elucidated because of the complexity of DN pathogenesis.

In traditional Korean medicine (TKM) and traditional Chinese medicine (TCM) theories, “wasting-thirst” (termed Sogal syndrome in TKM and Xiaoke syndrome in TCM) probably equate to the term “T2DM” in Western medicine (Xu et al., 2016). This syndrome refers to a Yin-deficient condition that causes heat in the body to increase, resulting in symptoms such as polydipsia, polyphagia, and polyuria as the disease progresses. The most serious condition of wasting-thirst syndrome is DN, which involves kidney problems and excessive urination.

Bekhogainsam decoction (BHID), created by Chang Chung-Ching in the Treatise on Febrile Disease (Shang Han Lun) is composed of Gypsum fibrosum (a mineral containing CaSO_4_ • 2 H_2_O), Anemarrhenae rhizome (the rhizome of *Anemarrhena asphodeloides* Bge.), Ginseng radix et rhizome (the root and rhizome of *Panax ginseng* C. A. Mey.), and Glycyrrhizae radix et rhizome (the root and rhizome of *Glycyrrhiza uralensis* Fisch. ex DC.) and Polished round-grained rice (the endosperm of *Oryza sativa* L. seeds) and is known to impact the lung and stomach through a unique effect of clearing heat and promoting fluid movement (Tong et al., 2012). The monarch, minister, assistant, and messenger medicines in TKM or TCM prescriptions reflect the overall concept of traditional medicine (TM). The effective action of TCM prescription is mediated through the whole function of the compatibility of medicines. In this prescription, Gypsum fibrosum is the monarch medicine, which is pungent and cold and relieves the heat of Qi. As a minister medicine, Anemarrhenae rhizoma is bitter, cold, smooth, and good at purging fire and nourishing Yin. The combination of the two components can exert a very strong effect on reducing heat, nourishing Yin, and moistening dryness. Ginseng radix et rhizoma is an assistant medicine that can nourish Qi and Yin. Glycyrrhizae radix et rhizoma and polished round-grained rice can be used as messenger medicines to replenish Qi, and to relieve fire without hurting the spleen and stomach. Given the compatibility of these medicines, BHID has strong clearing effects on heat, relieves fidgetiness, and nourishes Qi generative fluid. BHID is also a commonly used prescription to treat patients with T2DM in traditional clinics. Despite the fact that BHID is a representative prescription for diabetes, little is known about its effects on diabetic complications, especially DN, or the underlying mechanisms of action.

Metabolomics is the qualitative and quantitative analysis of metabolites with a molecular mass below 1,000 in the *in vivo* study using high-throughput instruments (Beger et al., 2016). It can be used to identify specific molecular markers in certain physiological and pathological conditions, and can be used to study the pathogenesis of metabolic diseases and the mechanism of action of therapeutic drugs (Barnes et al., 2016). With regard to the pathogenesis of T2DM treated with TM theories based on metabolomics (Cui et al., 2018; Lin et al., 2019), some studies have been reported that act as good examples for the present study.

Intestinal flora is the most stable of the colonizing microorganisms in the human body. With the continuous development of DNA sequencing, metabolomics, proteomics, and computer technology, research on microbial flora has expanded, and the mystery of microbial flora has gradually been uncovered (Zhang B. et al., 2019). Recent studies have found that intestinal flora can significantly regulate the secretion of insulin, glucagon, and other hormones, and play an important role in the development of insulin resistance (Hanning and Diaz-Sanchez, 2015; Horie et al., 2017; Barko et al., 2018).

Network pharmacology is a new subject based on the theory of systems biology (Yuan et al., 2017). Specific signal nodes are selected for multi-target drug molecular design through network analysis of biological systems. Network pharmacology emphasizes the regulation of multiple pathways of signal pathways to improve the therapeutic effect of drugs and reduce the toxic and side effects, conferring improvements in the success rate of clinical trials of new drugs and reduce the drug development costs. Owing to the complexity of traditional medicine prescriptions, the pharmacological mechanisms of the anti-DM or anti-DN actions are difficult to clarify. In accordance with the modern pharmacodynamic mechanism of TM, the current “one target and one drug” mode can be transferred to a new “network target and multicomponent” mode based on network pharmacology (Zhang et al., 2019). Network pharmacology is also one of the new methods and focus points of TM modern research, and provides an abundance of information about single herbs or the prescriptions of potential bioactive components and the active mechanisms at the molecular and systematic level.

Therefore, in the present study, we investigated the effects of BHID in mice with streptozotocin (STZ)-induced DN, along with the underlying mechanism; in particular, we focused on renal dysfunction through the assessment of metabolomics, gut microbiota, and network pharmacology based on the perspective of systems biology, to reveal the modern scientific interpretation of BHID in the treatment of DM and DN.

## Material and Methods

### Preparation of the BHID Extract and Quality Control

The characteristics of constituent drugs in BHID, based on traditional prescription theory, were presented in **Table 1**. All constituent drugs in BHID in **Table 1** were purchased from Medicinal Materials Company (Kwangmyungdang Medicinal Herbs, Ulsan, Korea). These species were authenticated by Prof. Xiangping Pei (Shanxi College of Traditional Chinese Medicine) before use. Voucher specimens (no. SXTCM-Meng-2019001 for Gypsum fibrosum; no. SXTCM-Meng-2019002 for Anemarrhenae rhizoma; SXTCM-Meng-2019003 for Ginseng radix et rhizoma; SXTCM-Meng-2019004 for Glycyrrhizae radix et rhizoma; SXTCM-Meng-2019005 for polished round-grained rice) were deposited in the Herbarium of Shanxi College of Traditional Chinese Medicine (SXTCM), Taiyuan, China. All drugs were mixed (total 190.52 g in **Table 1**), ground, and extracted with 1.9052 L of boiling water for 3 h. The extract was then filtered through Whatman No. 1 filter paper (GE Healthcare UK Limited, UK), concentrated under a vacuum rotary evaporator, and lyophilized in a freeze-dryer (II Shin BioBase Co., Yangju, Korea). The BHID extract (yield = 33.99%) was stored at 4°C until use; it was then dissolved in distilled water for use.

**Table 1:** Composition of Bekhogainsam decoction (BHID) and the characteristics of each constituent drugs.

Drug name/English name	Scientific name (Family)	Medicinal part	Weight (g) in BHID	Channel tropism	Medicinal efficacies	Role in the prescription
Gypsum fibrosum	–	Gypsum(mineral)	75	lung, stomach	Clear heat, purge fire, relieve agitation, stop thirst, heal wounds, and promote tissue regeneration	Monarch
Anemarrhenae rhizoma	*Anemarrhena asphodeloides* Bge.	Rhizome	30	lung, stomach, kidney	Clear heat and purge fire, generate fluids, and moisten dryness	Minister
Ginseng radix et rhizoma.	*Panax ginseng* C. A. Mey.	Root, Rhizome	15	lung, spleen, heart	Replenish the primordial qi, tonify spleen and lung, promote fluid production, and induce tranquilization	Assistant
Glycyrrhizae radix et rhizoma	*Glycyrrhiza uralensis* Fisch. ex DC.	Root, Rhizome	10.52	heart, lung, spleen, stomach	Tonify spleen and replenish qi, dispel phlegm and arrest cough, relieve spasm and pain, clear heart and relieve toxicity, harmonize all medicines	Messenger
Polished round-grained rice	*Oryza sativa* L	Endosperm of seeds	60	spleen, stomach, lung	Invigorate qi and strengthen the spleen, relieve polydipsia	Messenger

In view of the different chemical structures that yield different pharmacodynamic effects, we performed quality control analysis of the BHID extract by using high-performance liquid chromatography (HPLC) under three different chromatographic conditions. The mobile phase for mangiferin and neo-mangiferin consisted of acetonitrile (A) and deionized water with 0.1% phosphoric acid (B). The gradient elution program was as follows: 3% A, 0–10 min; 15% A, 10–25 min. The detection wavelength was set at 258 nm. The mobile phase for liquiritin and ammonium glycyrrhetate consisted of acetonitrile (A) and deionized water with 0.1% phosphoric acid (B). The gradient elution program was as follows: 19% A, 0–8 min; 19%–50% A from 8–35 min; 50%–100% A from 35–36 min; then 100%–19% A from 36–40 min. The detection wavelength was set at 237 nm. The mobile phase for ginsenoside-Rg1 consisted of acetonitrile (A) and deionized water with 0.1% phosphoric acid (B), and the isocratic elution program was as follows: 25% A at 0–25 min. The detection wavelength was set at 218 nm. All HPLC analyses were performed with a U3000 series system (Thermo Fisher Scientific, Waltham, MA, USA) and samples were separated on a Tnature C18 column (250 × 4.6 mm, 5 µm, Waters, Milford, MA, USA). The HPLC pattern of components in BHID water extract has been reported elsewhere (Ding et al., 2007; Ablat et al., 2015; Song et al., 2015). The flow rate was 1.0 ml/min, the injection volume was 10 µl, and the column temperature was maintained at 30°C. Liquiritin, ammonium glycyrrhetate, mangiferin, neo-mangiferin, and ginsenoside-Rg1 standards (production batch numbers 18060604, 18083102, 18051002, 18041112, and 18071601, respectively) were purchased from Chengdu Pufei De Biotech Co., Ltd. (Chengdu, Sichuan, China). The mass fractions of all standard reagents were ≥ 98%.

### Animals and Preparation of Diabetic Animal Model

Five-week-old male specific pathogen-free (SPF) C57BL/6 mice (body weight 18–19 g) were purchased from Orient Bio Inc. (Seongnam, Korea). The mice were permitted a 1-week acclimation period to the SPF laboratory conditions (IVC systems, LAB & BIO, Changwon, Korea) before the experiments were started. The mice were kept under a 12 h/12 h light/dark cycle, at 23°C±2°C, with 50%±10% humidity, and were administered sterile filtered water, and used corn cobs as bedding. All animals were handled in accordance with the animal welfare guidelines issued by the Korean National Institute of Health and the Korean Academy of Medical Sciences for the care and use of laboratory animals and approved by the Institutional Animal Care and Use Committee of Dongguk University (IACUC-2017-012).

After adaptive breeding, all mice were randomly divided into five groups (*n*=7 per group): the normal group; the STZ-induced diabetic control group; the low-dose BHID treatment group, the high-dose BHID treatment group, and the metformin-treated group. With the exception of the mice in the normal group, DN was induced in all mice by STZ intraperitoneal injections at 100 mg/kg of body weight (b.w.) (Sigma-Aldrich, St. Louis, MO, USA) once per day for 3 days consecutively (Glastras et al., 2016). Animals with blood glucose concentrations of >300 mg/dl were used for the study. Mice in the low- and high-dose BHID treatment groups were administered 100 and 500 mg/kg b.w. BHID (peroral injection, p.o.), and mice in the metformin-administered group were treated with 250 mg/kg metformin (p.o.). The mice in the normal and STZ-induced diabetic control groups were treated with an equal volume of saline at the same time. All drugs were administered orally once per day for 4 weeks. All mice were administered a sterile standard diet (3.1 kcal, 14% protein, Harlan Teklad, Madison, WI, USA) for the entire experimental period. After a 4-week administration period, all mice were fasted for 12 h and then sacrificed. Samples of the blood, feces, and kidneys were harvested for further analysis on a clean bench. Subsequently, the intestinal feces were collected under sterile conditions, and then placed into sterile tubes and stored in liquid nitrogen.

### Measurement of Physiological Characteristics

Changes in physiological parameters, such as body weight, water, food intake, and fasting blood glucose (FBG) levels, were measured once per week for 4 weeks. In addition, blood samples were collected by cutting the tip of the tails, and 24 h urine volumes were collected in the metabolic cages from all mice at the end of the experimental period.

### Measurement of Serological and Urine Markers

A commercial ELISA kit (Crystal Chem, Springfield, IL, USA) was used to measure the insulin concentration in the serum in accordance with the manufacturer’s protocol. The levels of aspartate aminotransferase (AST), alanine aminotransferase (ALT), glucose, total cholesterol (TC), triglyceride (TG), HDL-cholesterol (HDL-C), urea nitrogen (UN), creatinine (Cr), and microalbumin/urine creatine (MA/UCREA) were measured in the serum or urine samples by using an automatic biochemistry analyzer (Fuji Dri-chem 700i, Fujifilm, Tokyo, Japan).

### Histopathological Observation

The kidney tissues were fixed in 4% paraformaldehyde in 0.1 M PBS, embedded in paraffin, and cut into 5-mm sections. To observe structural damage to renal tissue, sections were stained with hematoxylin and eosin (H&E), periodic acid-Schiff (PAS), and Masson’s trichrome (M-T). The sections were visualized using light microscopy, and digital images were captured and analyzed.

### Metabolomic Profiling

Serum samples were thawed at 4°C on ice. Then, 100 μl of sample was taken and placed in an EP tube, extracted with 400 μl of extraction solvent (methanol: acetonitrile = 1:1 [v/v], containing an internal standard, L-2 chlorophenylalanine at 2 μg/ml), vortexed for 30 s, treated with ultrasound for 5 min (during incubation in ice water), and incubated for 1 h at -20°C to precipitate proteins. The solutions were then centrifuged at 12,000 rpm for 15 min at 4°C. The supernatant was transferred into fresh EP tubes, and the extracts were dried in a vacuum concentrator without heating, and reconstituted in 200 μl of the extraction solvent (acetonitrile: water = 1:1 [v/v]). The reconstituted solution was vortexed for 30 s and sonicated for 10 min (in a 4°C water bath), centrifuged for 15 min at 12,000 rpm at 4°C. The supernatant was transferred into a fresh 2 ml LC-MS glass vial, 10 μl aliquots from each sample were collected and pooled as QC samples, and the supernatant was collected for UHPLC-QTOF-MS analysis.

LC-MS/MS analyses were performed using a UHPLC system (Thermo Fisher Scientific, Waltham, MA, USA) with a Thermo C18 column (1.9 μm; 2.1 × 50 mm). The mobile phase consisted of acetonitrile (A) and deionized water with 0.1% methanoic acid (B), and the following elution gradient was used: 0–1 min, 5%–5% A; 1–5 min, 5%–15% A; 5–7 min, 15%–15% A; 7–14 min, 15%–100% A; 14–19 min, 100%–100% A; 19–20 min, 100%–5% A; 20–22 min, 5%–5% A. The mobile phase was delivered at 0.3 ml min^-1^, and the injection volume was 3 μl.

The Triple TOF mass spectrometer was used for its ability to acquire MS/MS spectra on a full MS dd-ms2 during the LC-MS experiment. In this mode, the electrospray capillary voltage was 3.2 kV in positive and negative ionization mode, the atomized gas velocity was 35 L·min^-1^, the auxiliary air velocity was 5 L·min^-1^, the ion source temperature was 320°C, and the auxiliary heater temperature was 350°C. The NCE values of energy collision were 25, 35, and 45, respectively.

The LC/Q/TOF-MS metabolomic profiling data were imported into Compound Discoverer 3.0 to perform the metabolic feature extraction by the adoption of a molecular feature extraction algorithm (Thermo Fisher, Inc., Santa Clara, CA, USA). The parameters were set as follows: mass range, 100–1,500; mass deviation, 5×10^-6^; retention time deviation, 0.05 min; SNR threshold, 3. SIMCA-P (Version 14.1, Umetrics AB, Umea, Sweden) was used for multivariate statistical analysis of the integral values obtained from LC-MS findings. The mean centered data were used for principle component analysis (PCA). The modeling of sample classes was performed using orthogonal projection to latent structure-discriminant analysis (OPLS-DA) algorithm at a unit variance-scaled modality. After the OPLS-DA test, only the integral, with variable importance in the projection (VIP) values of >1.5 and a p-value of <0.05 from Student’s *t*-test, was considered to be the potential differential metabolite. The disturbed metabolites and metabolic pathways were identified by open database sources, including the Human Metabolome Database^1^, KEGG^2^, and MetaboAnalyst^3^. Heat maps were constructed using Pyplot^4^(Version 2.0.2).

### Gut Microbiota Analysis

Mouse fecal samples were randomly selected from each group and thawed at 4°C on ice. The total microbial DNA was extracted by using a commercial DNA kit (OmegaBio-tek, Norcross, GA, USA) in accordance with the manufacturer’s protocol. The primers were obtained according to the conservative region design, and the end of the primers was amended with a sequencing connector to amplify the bacterial V3–V4 region of the 16S rRNA gene. Polymerase chain reaction (PCR) amplification was conducted, and the PCR products were extracted from 1% agarose gel, and purified using the AxyPrep DNA Gel Extraction Kit (Axygen Biosciences, Union City, CA, USA) in accordance with the manufacturer’s instructions and quantified using a Quantus™ fluorometer (Promega, Madison, WI, USA). The purified amplicons were sequenced using an Illumina HiSeq 2500 (Illumina, San Diego, CA, USA) in accordance with the manufacturer’s guidelines. Alpha diversity analysis, operational taxonomic units (OTUs) clustering analysis, and beta diversity analysis were conducted successively.

### Network Pharmacology Profiling

Chemical ingredients and the targets of BHID were obtained from the Traditional Chinese Medicine Systems Pharmacology database^5^ (TCMSP), a Bioinformatics Analysis Tool for Molecular mechANism of Traditional Chinese Medicine^6^ (BATMAN-TCM), and the relevant literature.

In consideration of the complexity of the changes in pharmacodynamic chemical components in the formula, the compounds were screened for both the pharmacokinetic and pharmacodynamic properties (oral bioavailability (OB) >30% and drug-likeness (DL) >0.18). The related targets of DN were collected from Genecards^7^ and the Online Mendelian Inheritance in Man (OMIM) database^8^, respectively. Venn diagram analysis was used for a comprehensive analysis of BHID, directly related genes, and targets of DN. A comprehensive, integrated analysis of BHID, directly related genes, and DN targets was performed using a Venn analysis. The overlap was presumed to be the group of potential targets, and these were used in the network pharmacology analysis. An interaction network was established among active ingredients, putative targets, and the DN-associated targets of BHID. The interaction network was visualized using Cytoscape 3.7.1 software^9^. STRING 11.0^10^ was chosen to establish the protein-protein interaction network (PPI network). The Database for Annotation, Visualization, and Integrated Discovery^11^ was used to perform Gene Ontology (GO) function enrichment analysis, and the Kyoto Encyclopedia of Genes and Genomes (KEGG)^2^ Pathway Database was used for pathway enrichment analysis of BHID.

### Quantitative PCR Assay

Total RNA was isolated by using NucleoZOL reagent (MACHEREY-NAGEL GmbH & Co.KG., Duren, Germany) in accordance with the manufacturer’s instructions. RNA concentration was measured using a spectrophotometer (NanoDrop Technologies, Inc., Wilmington, DE, USA). cDNA was generated with 1 μg of total RNA using a Reverse Transcription System kit (Promega, Fitchburg, WI, USA). PCR was performed using a SYBR Green kit (Agilent Technologies, West Cedar Creek, TX, USA) and primers specific to the target genes, namely: PKC-α: 5′-CCCATTCCAGAAGGAGATGA-3′ (forward, accession no. NM_011101.3) and 5′-TTCCTGTCAGCAAGCATCAC-3′ (reverse, accession no. NM_011101.3); α-SMA: 5′-ACTGCCGAGCGTGAGATTGT-3′ (forward, accession no. XM_006526606.2) and 5′-TGATGCTGTTATAGGTGGTTTCG-3′ (reverse, accession no. XM_006526606.2); TGF-β1: 5′-CGAAGCGGACTACTATGCTAAAGAG-3′ (forward, accession no. NM_011577.2) and 5′-TGGTTTTCTCATAGATGGCGTTG-3′ (reverse, accession no. NM_011577.2); GAPDH: 5′-CAGCCTCGTCCCGTAGACA-3′ (forward, accession no. XM_017321385.1) and 5′-CGCTCCTGGAAGATGGTGAT-3′ (reverse, accession no. XM_017321385.1). Gene expression was evaluated by the ΔΔCT method with GAPDH as a housekeeping gene.

### Western Blotting Analysis

Kidney tissues were isolated by using T-PER tissue protein extraction reagent (Thermo Fisher Scientific, Waltham, MA, USA). The protein content was measured using a Bradford assay, separated using SDS-PAGE and transferred to a nitrocellulose membrane. The membranes were incubated in 5% skimmed milk, dissolved in Tris-buffered saline with 1% Tween-20 (TBST), pH 7.5, for 30 min at RT for blocking, and then incubated with anti-PKCα, I-κB, NF-κB (p 65), α-SMA, TGF-β1, COX-2 (Santa Cruz Biotechnology, Santa Cruz, Dallas, TX, USA), iNOS (BD Biosciences, San Jose, CA, USA), total-AKT (Ser473), phospho-AKT (Ser473), total-IRS (Ser 307), phospho-IRS (Ser 307), PI3K (p85) (Cell Signaling Technology, Danvers, MA, USA), and β-actin (Sigma-Aldrich, Inc., St. Louis, MO, USA) antibodies at 1:1,000 overnight at 4°C and incubated with HRP-conjugated mouse or rabbit secondary antibodies for 3 h at RT. Blots were scanned and analysed with ChemiDoc^TM^ MP imaging system.

### Statistical Analysis

Data analyses were conducted using GraphPad Instat software (ver. 5.0; GraphPad Software, La Jolla, CA, USA). The data were expressed as the mean ± standard deviation (SD) of seven mice in each group. The significance of treatment effects was determined by one-way analysis of variance (ANOVA) followed by Tukey’s post-hoc analysis. The p-value of 0.05 was considered to indicate statistical significance.

## Results

### HPLC Profiles of BHID

**Figure 1:**
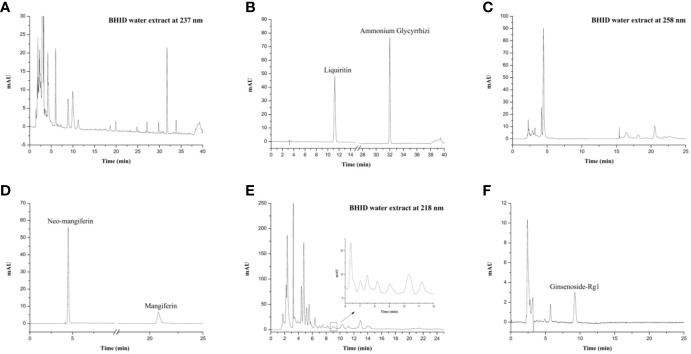
High-performance liquid chromatography (HPLC) profiles of Bekhogainsam decoction (BHID). HPLC patterns of the BHID extract at 237 nm **(A)**, 258 nm **(B)**, and 218 nm **(E)**, and the standard compounds, liquiritin, ammonium glycyrrhetate, mangiferin, neo-mangiferin, and ginsenoside-Rg1 at 237 nm **(B)**, 258 nm **(D)**, and 218 nm **(F)**.

As shown in **Figure 1**, the HPLC profiles of BHID contained peaks reflecting the main compounds: mangiferin and neo-mangiferin in Anemarrhenae rhizome; liquiritin and ammonium glycyrrhetate in Glycyrrhizae radix et rhizome; and ginsenoside-Rg1 in Ginseng radix et rhizoma. The equations of the calibration curves for these reference compounds were Y=131.48X–0.0552 (r=0.9992) for liquiritin, Y=43.268X+0.3934 (r=0.9991) for ammonium glycyrrhetate, Y=643.55X+0.0189 (r=0.9998) for mangiferin, Y=351.79X+2.4417 (r=0.9991) for neo-mangiferin, and Y=4.4811X+0.0258 (r=0.9999) for ginsenoside-Rg1; the content determination of the five compounds in BHID are shown in **Table 2**.

**Table 2:** Quantification of Bekhogainsam decoction (BHID) by high-performance liquid chromatography (HPLC) analysis.

Component name	Ratio (mg/g)
Liquiritin	0.123±6.54×10^-4^
Ammonium glycyrrhetate	1.14±7.88×10^-3^
Mangiferin	0.186±4.07×10^-3^
Neo-mangiferin	1.103±1.09×10^-2^
Ginsenoside-Rg1	0.110±2.16×10^-2^

### Effects of BHID on Physiological and Serological Changes in Diabetic Nephropathy Mice

**Figure 2:**
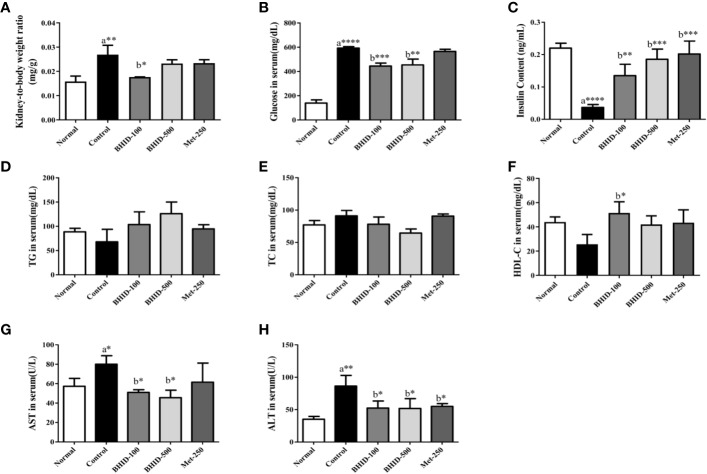
Effects of Bekhogainsam decoction (BHID) on physiological and serological changes in mice with diabetic nephropathy (DN). Kidney to body weight ratio **(A)** and serum levels of glucose **(B)**, insulin **(C)**, triglyceride (TG) **(D)**, total cholesterol (TC) **(E)**, HDL-cholesterol (HDL-C) **(F)**, aspartate aminotransferase (AST) **(G)**, and alanine aminotransferase (ALT) **(H)**. Normal, normal group; Control, streptozotocin (STZ)–induced DN control group; BHID-100, BHID 100 mg/kg treatment group; BHID-500, BHID 500 mg/kg treatment group; and Met-250, Metformin 250 mg/kg treatment group. Data are expressed as the mean ± standard deviation (SD) (*n*=7 per group); *P < 0.05, **P < 0.01, and ***P < 0.001 , ****P < 0.0001 vs. normal (a) or control (b) groups.

Over 4 weeks, the administration of BHID at low (100 mg/kg) and high (500 mg/kg) doses significantly reduced the increase in food intake (*p*<0.0001 and *p*<0.001, respectively) and water (*p*<0.0001, respectively) intake compared with the DN control group (**Table 3**). Moreover, the administration of BHID significantly increased body weight in weeks 3 (*p*<0.01 for low-dose treatment and *p*<0.05 for high-dose treatment, respectively) and 4 (*p*<0.05 for low-dose) compared with the DN control group (**Table 3**). In addition, low doses of BHID significantly increased the ratio of the kidney to the body weight compared with the DN control group (*p*<0.05) after administration for 4 weeks (**Figure 2A**). The administration of high doses of BHID and metformin also resulted in the same trend, but without significant differences.

**Table 3:** Food intake, water intake, body weight, and fasting blood glucose (FBG) parameters of mice with streptozotocin (STZ)–induced diabetic nephropathy (DN) treated with Bekhogainsam decoction (BHID).

	Weeks	Normal	Control	BHID-100	BHID-500	Met-250
Food intake (g)	1	18.43±0.73	74.43±2.87^a****^	71.14±0.64	71.14±0.83	72.29±1.39
	2	30.57±1.59	68.29±1.28^a****^	47.40±7.23^b****^	49.25±11.50^b****^	44.86±12.02^b****^
	3	26.29±1.39	74.14±3.94^a****^	54.00±3.35^b****^	57.00±12.53^b***^	60.86±2.29^b***^
	4	18.71±2.37	63.50±2.69^a****^	43.00±1.41^b****^	43.50±13.16^b****^	49.83±2.73^b**^
Water intake (ml)	1	29.43±0.90	289.29±11.16^a****^	238.29±4.37^b**^	172.83±45.88^b****^	194.29±5.60^b****^
	2	29.71±2.76	288.57±11.25^a****^	162.00±35.44^b****^	150.00±46.90^b****^	200.00±15.12^b****^
	3	31.86±2.80	261.43±22.95^a****^	157.00±24.00^b****^	180.00±50.87^b****^	215.00±21.88^b**^
	4	31.14±2.53	261.67±36.70^a****^	170.00±10.80^b****^	141.25±50.05^b****^	236.67±4.71
Bodyweight (g)	1	17.86±1.36	17.86±1.96^a****^	17.43±1.99	16.40±2.58	17.14±1.46
	2	25.29±1.75	18.86±1.81^a****^	18.60±3.20	19.00±1.22	18.14±1.73
	3	24.86±1.36	14.80±1.17^a****^	19.00±3.16^b*^	19.75±1.92^b**^	19.00±1.77^b*^
	4	24.57±0.73	15.75±1.09^a****^	20.33±2.36^b*^	20.00±1.63	19.20±1.94
FBG (mg/dl)	1	151.29±6.30	490.29±12.43^a****^	476.29±46.96	470.17±17.53	493.00±10.05
	2	160.71±7.59	507.43±18.39^a****^	444.00±81.39	449.00±26.42	508.33±25.85
	3	141.57±17.88	420.86±83.13^a****^	189.50±90.50^b****^	242.33±68.39^b***^	478.43±49.47
	4	152.25±21.60	574.67±36.39^a****^	472.00±21.31	478.00±128.08	407.20±88.82^b****^

Normal, normal group; Control, STZ-induced DN control group; BHID-100, BHID 100 mg/kg treatment group; BHID-500, BHID 500 mg/kg treatment group; and Met-250, Metformin 250 mg/kg treatment group. Data are expressed as the mean ± S.D. (n=7 per group); *P < 0.05, **P < 0.01, and ***P < 0.001, ****P < 0.0001 vs. normal (a) or control (b) groups.

As shown in **Table 3**, an increase in FBG levels in the DN control group (*p*<0.0001 throughout the experimental period) was significantly reduced by BHID administration (*p*<0.0001 for low-dose treatment and *p*<0.001 for high-dose treatment in week 3) and metformin (*p*<0.0001 in week 4). As shown in **Figure 2B**, BHID also reduced the elevated levels of serum glucose at both low (*p*<0.001) and high (*p*<0.01) doses. Moreover, the BHID treatment groups showed a greater glucose lowering effect than 250 mg/kg metformin. As shown in **Figure 2C**, BHID administration induced significant increases in serum insulin levels at low (*p*<0.01) and high (*p*<0.001) doses compared with the control group (**Figure 2C**). Metformin also significantly decreased insulin levels (*p*<0.001). The levels of triglyceride (TG, **Figure 2D**) and total cholesterol (TC, **Figure 2E**) were not significantly changed by the administration of BHID, whereas the levels of HDL-cholesterol (HDL-C, **Figure 2F**) were significantly higher in the low-dose BHID treatment group. In addition, the serum levels of AST (**Figure 2G**) and ALT (**Figure 2H**) were increased significantly (*p*<0.05 for AST and *p*<0.01 for ALT) in the control group compared with the normal group, and these increases were reduced significantly by BHID administration in both the low-dose (*p*<0.05 for AST and *p*<0.05 for ALT) and high-dose (*p*<0.05 for AST and *p*<0.05 for ALT) treatment group compared with the control group. Metformin induced a significant reduction in ALT levels (*p*<0.05).

### Effects of BHID on Kidney Dysfunction in Mice With DN

**Figure 3:**
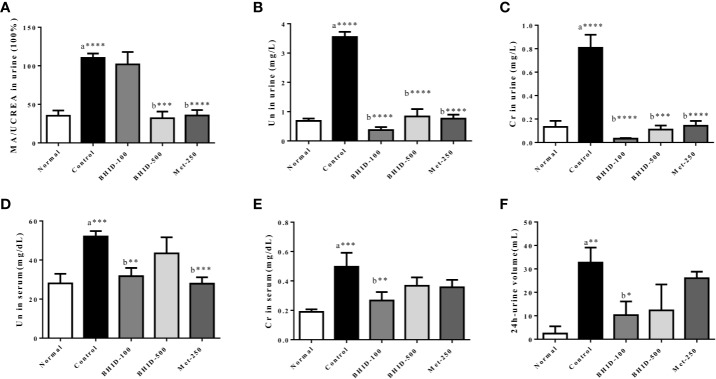
Effects of Bekhogainsam decoction (BHID) on kidney dysfunction in mice with diabetic nephropathy (DN). The urine levels of microalbumin/urine creatine (MA/UCREA) **(A)**, urea nitrogen (UN) **(B)**, createnine (Cr) **(C)**, and UN **(D)**, serum levels of Cr **(E)**, and urine volumes for 24 h at week 4 **(F)**. Normal, normal group; Control, streptozotocin (STZ)–induced DN control group; BHID-100, BHID 100 mg/kg treatment group; BHID-500, BHID 500 mg/kg treatment group; and Met-250, Metformin 250 mg/kg treatment group. The data are expressed as the mean ± standard deviation (*n*=7 per group). *P < 0.05, **P < 0.01, and ***P < 0.001, ****P < 0.0001 vs. normal (a) or control (b) group.

As shown in **Figure 3A**, a significant increase in MA/UCREA was observed in the DN control group compared with the normal group. The administration of BHID (*p*<0.001 for high dose) or metformin (*p*<0.0001) for 4 weeks significantly decreased MA/UCREA levels relative to the control group. In addition, the BHID-treated groups showed significant decreases in urea nitrogen (**Figure 3B**) and creatinine (**Figure 3C**) levels with low-dose (*p*<0.0001) and high-dose (*p*<0.0001 for urea nitrogen and *p*<0.001 for creatinine, respectively) treatment, respectively. Metformin also led to significant decreases in urea nitrogen (*p*<0.0001) and creatinine (*p*<0.0001). BHID and metformin also significantly reduced the increasing levels (*p*<0.01 for low-dose BHID and *p*<0.001 for metformin treatment) of urea nitrogen (**Figure 3D**) and creatinine (*p*<0.01 for low-dose BHID treatment, **Figure 3E**) in the sera of mice with DN. Finally, a significant decrease in 24 h urine volume (*p*<0.05) was observed in the group administered low-dose BHID (**Figure 3F**).

### Effects of BHID on Histopathologic Changes in the Kidney Tissue of Mice With DN

**Figure 4:**
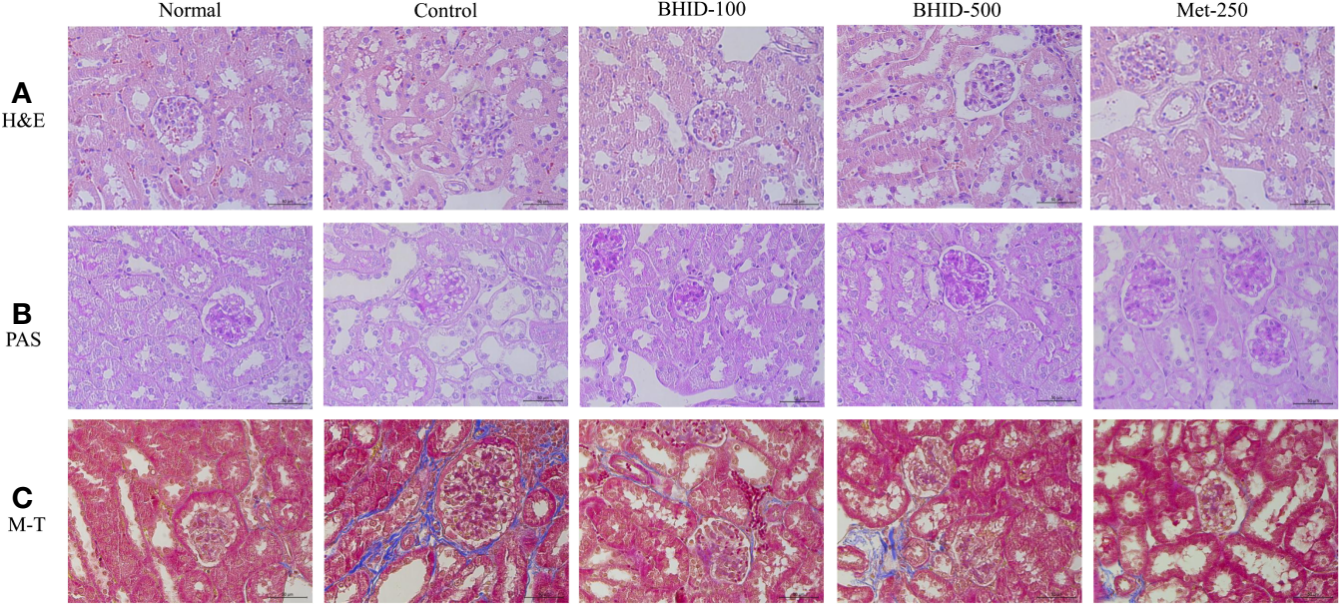
Effects of Bekhogainsam decoction (BHID) on histological changes in kidney tissues in mice with diabetic nephropathy (DN). Tissue staining with hematoxylin and eosin (H&E) **(A)**, periodic acid-Schiff (PAS) **(B)**, and Masson’s trichrome (M-T) **(C)**. Normal, normal group; Control, streptozotocin (STZ)–induced DN control group; BHID-100, BHID 100 mg/kg treatment group; BHID-500, BHID 500 mg/kg treatment group; and Met-250, Metformin 250 mg/kg treatment group. All tissues were observed using a light microscope; representative images are shown (×400).

The effects of BHID on histopathological changes in the kidney of mice with DN are shown in **Figure 4**. The kidney tissues were stained with H&E, PAS, and M-T. Hematoxylin and eosin staining revealed well-opened glomerular capillary loops in the normal group. In the DN control group, morphological changes, including mesangial hyperplasia, irregular distortions, and glomerular basement membrane thickening, were observed. However, histological changes in glomerular and tubular structures were, to some extent, prevented in both the BHID-treatment groups and the metformin treatment group. PAS clearly revealed increased thickness of the glomerular basement membrane (GBM), hypertrophy, and mesangial matrix expansion in the control group. M-T staining also revealed an increased intensity of fibrosis in response to accumulation of extracellular matrix proteins in the control group. However, all destructive changes in mice with DN were ameliorated markedly by administration of both the low and high doses of BHID and metformin.

### Effects of BHID on the Expression of Fibrosis Regulators in Renal Tissues of Diabetic Nephropathy Mice

**Figure 5:**
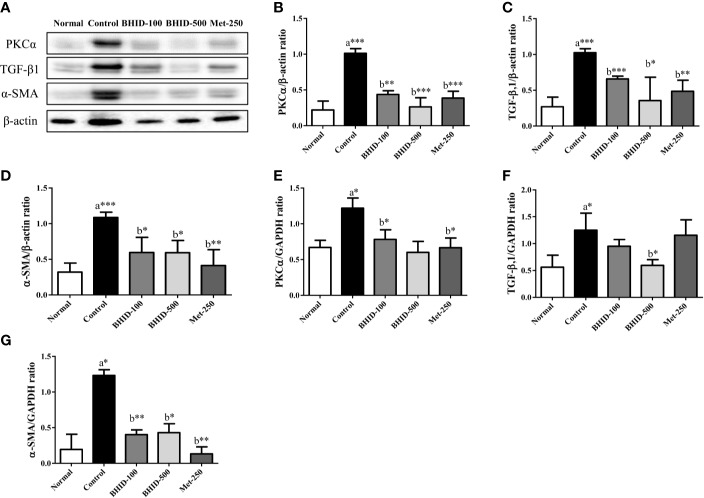
Effects of Bekhogainsam decoction (BHID) on the expression of PKCα, TGF-β1, and α-SMA in the kidney tissue of mice with diabetic nephropathy (DN). Expression of PKCα, TGF-β1, and α-SMA was determined by western blotting **(A**–**D)** and qPCR **(E**–**G)**, respectively. Relative expression of PKCα **(B**, **E)**, TGF-β1 **(C**, **F)**, and α-SMA **(D**, **G)** were calculated using β-actin or GAPDH for normalization. Normal, normal group; Control, streptozotocin (STZ)–induced DN control group; BHID-100, BHID 100 mg/kg treatment group; BHID-500, BHID 500 mg/kg treatment group; and Met-250, Metformin 250 mg/kg treatment group. The data are expressed as the mean ± standard deviation (*n*=7). *P<0.05, **P<0.01, and ***P<0.001 vs. normal (a) or control (b) group.

To investigate the effects of BHID on renal fibrosis in DN, we measured the mRNA and protein expression of renal fibrosis regulators (PKCα, TGF-β1, and α-SMA) by qPCR and western blotting (**Figure 5A**), respectively. The expression of PKC-α (**Figures 5B, E**) and TGF-β1 (**Figures 5C, F**) in kidney tissues was significantly increased in the DN control group compared with the normal group (*p*<0.001 for protein, *p*<0.05 for mRNA for both regulators). The expression of PKCα in kidney tissues of mice with DN was attenuated by the administration of low-dose (*p*<0.01 for protein and *p* <0.05 for mRNA) and high-dose (*p*<0.001 for protein) BHID as well as by metformin (*p*<0.001 for mRNA and *p*<0.05 for protein). We also found that the expression of TGF-β1 was increased significantly (*p*<0.001 for protein and *p*<0.05 for mRNA) in the control group relative to the normal group (**Figures 5C, F**). BHID administration significantly inhibited TGF-β1 protein (*p*<0.001 for low dose and *p*<0.05 for high dose) and mRNA (*p*<0.05 for high dose) expression compared with the control group. Metformin also significantly decreased TGF-β1 protein expression (*p*<0.01).

A significant increase in α-SMA protein (*p*<0.001, **Figure 5D**) and mRNA (*p*<0.05, **Figure 5G**) expression was observed in the control group relative to the normal group. The administration of BHID significantly decreased the expression of α-SMA protein (*p*<0.05 for low and high dose) and mRNA (*p*<0.01 for low dose and *p*<0.05 for high dose) compared with the control group. Metformin administration also significantly inhibited α-SMA expression (*p*<0.01 for mRNA and protein).

### Differential Metabolite Analysis of Serum

In the present study, high-dose (500 mg/kg) BHID improved the symptoms of mice with STZ-induced DN. Therefore, the high-dose BHID was selected as the optimal effective dose for metabolic analysis and used in the subsequent screening for metabolic differences. The metabolite profiles of all serum samples were analyzed to determine the relative levels of the metabolites based on UHPLC-Q/TOF-MS analyses, and the base peak intensity chromatograms of a representative serum sample in each group are shown in **Supplementary Figure 1**. In the positive ion mode, more components were detected, and the multivariate statistical analysis was dependent on this mode.

**Figure 6:**
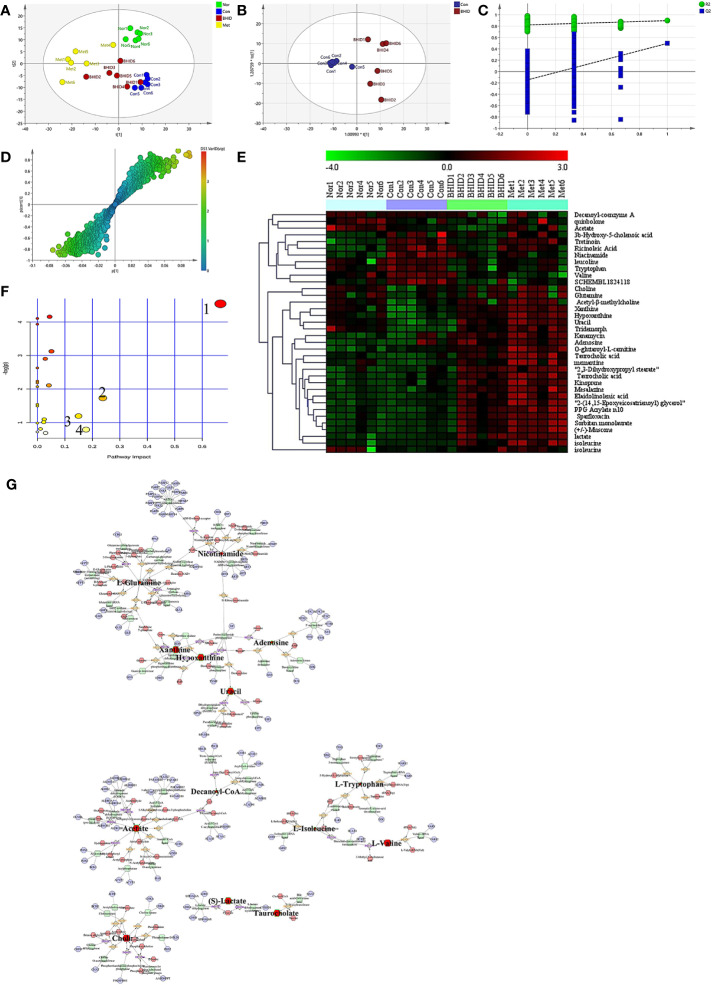
Differential metabolite analysis of serum. The principle component analysis (PCA) analysis in different groups **(A)** and orthogonal projection to latent structure-discriminant analysis (OPLS-DA) analysis between control and Bekhogainsam decoction (BHID)–treatment group **(B)**. The permutation test of the OPLS-DA mode between the control and BHID groups **(C)** and the S-plot analysis **(D)**. The heat map of potential metabolites **(E)** and the summary of pathway analysis of differential metabolites **(F)**: valine, leucine, and isoleucine biosynthesis (1); nicotinate and nicotinamide metabolism (2); tryptophan metabolism (3); and alanine, aspartate, and glutamate metabolism (4). Metabolite-reaction–enzyme-gene graph **(G)**. The hub metabolites are presented in bold font. Nor, normal group; Con, streptozotocin (STZ)–induced DN control group; BHID, BHID 500 mg/kg group; and Met, metformin 250 mg/kg group.

First, PCA was conducted on serum samples. Clear differences in the serum metabolome were observed among all four groups (**Figure 6A**), especially between the control and other groups. The BHID and metformin group samples more closely resembled the normal group than the control group.

OPLS-DA and S-plot were then performed and compared to identify and characterize metabolites (**Figures 6B–D**). The significant differences in serum samples were observed between the control and BHID groups (R^2^Y=0.822, Q^2^=-0.146). There were 36 specific metabolic biomarkers that distinguished between the BHID and control group, including: xanthine, hypoxanthine, mesalazine, kanamycin, acetyl-β-methylcholine, uracil, taurocholic acid, elaidolinolenic acid, choline, adenosine, PPG acrylate n10, 2,3-dihydroxypropyl stearate, kinoprene, 2-(14,15-epoxyeicosatrienoyl) glycerol, tridemorph, lactate, quinbolone, memantine, sorbitan monolaurate, sparfloxacin, 3b-hydroxy-5-cholenoic acid, O-glutaroyl-L-carnitine, (+/-)-muscone, tretinoin, ricinoleic acid, niacinamide, leucoline, decanoyl-coenzyme A, tryptophan, isoleucine, glutamine, valine, acetate, SCHEMBL1824118, and isoleucine. The heat map for these metabolites in the BHID and control groups is shown in **Figure 6E**. These data points were clustered into four distinct groups in the plot map, with a clear separation of the normal, control, BHID, and metformin samples.

The biomarkers that were altered in the BHID group were identified to be involved in four main pathways based on metabolic pathway analysis with MetaboAnalyst 4.0. The impact values of (a) valine, leucine, and isoleucine biosynthesis; (b) nicotinate and nicotinamide metabolism; (c) tryptophan metabolism; (d) alanine, aspartate, and glutamate metabolism were 0.67, 0.24, 0.18, and 0.15, respectively, and those metabolic pathway were the key metabolites in each pathway (**Figure 6F**).

The key metabolites from the metabolic pathway analysis were loaded into MetScape, and a metabolite–reaction–enzyme–gene graph was established to obtain an overview of all BHID metabolites on DN (**Figure 6G**). Adenosine, hypoxanthine, L-glutamine, uracil, xanthine, nicotinamide, acetate, decanoyl-Co, L-isoleucine, L-tryptophan, L-valine, (S)-lactate, taurocholate, and choline were selected as the hub metabolites of the correlation network in BHID on DN, based on their degree of influence.

### Gut Microbiota Analysis

**Figure 7:**
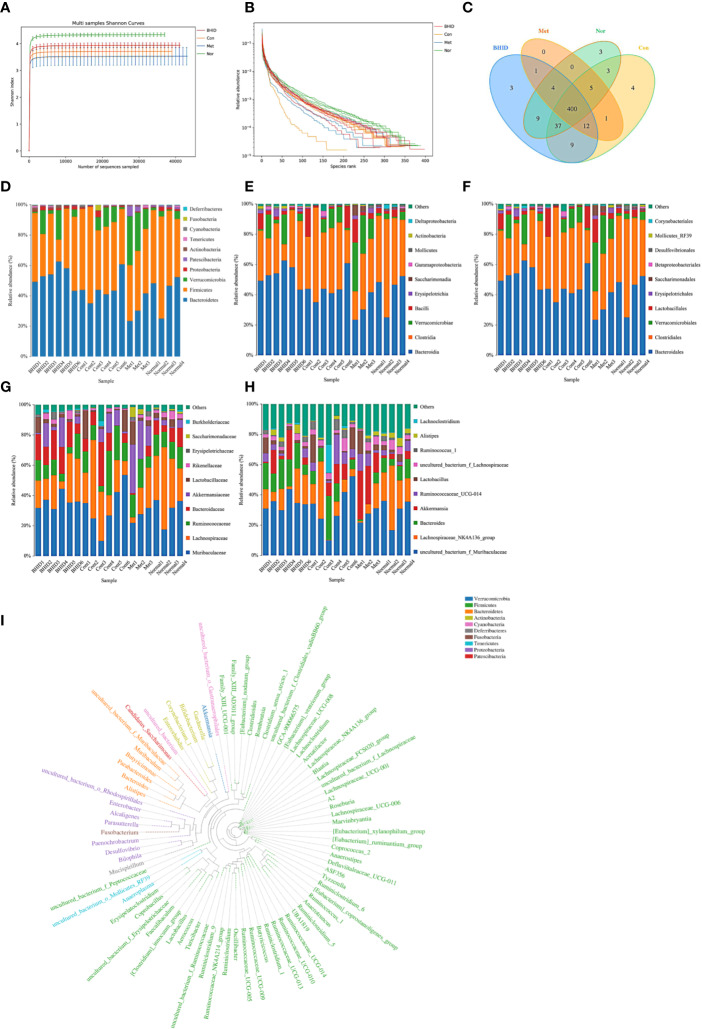
Overall structural modulation of gut microbiota. Shannon curves for all groups **(A)** and rank curves **(B)**. Shared and special operational taxonomic unit (OTUs) for the normal, control, Bekhogainsam decoction (BHID), and metformin groups **(C)**. The information of phylum, class, order, family, and genus level for each sample **(D–H**). The phylogenetic tree of OTUs at the genus taxonomic level **(I)**. Nor, normal group; Con, streptozotocin (STZ)–induced diabetic nephropathy (DN) control group; BHID, BHID 500 mg/kg group; and Met, metformin 250 mg/kg group.

The Shannon curves (**Figure 7A**) and Rank curves (**Figure 7B**) of each group were relatively gentle and concentrated, indicating that the amount of sequencing data was sufficient to reflect the majority of microbial information in the samples. The Shannon value provides an indication of the diversity of the intestinal flora in the samples (**Figure 7A**). The abscissa represents the number of sequences and the ordinate represents the Shannon index value. The higher the community diversity, the greater the Shannon value. The richness of intestinal flora and evenness were shown by the Rank abundance curves (**Figure 7B**). The length of the abscissa reflects the richness of the intestinal flora. The wider the curve, the richer the composition. The uniformity of the composition of the flora was reflected by the shape of the curve: a flatter curve indicates a more uniform composition.

OTUs at the 97% similarity level were obtained by cluster tags, and taxonomy notes were made on OTUs based on the taxonomy databases of Silva (for bacteria) and UNITE (for fungi). After the intersection was prepared using a OTUs-Venn diagram, we found that there were 400 shared OTUs for four groups (**Figure 7C**); the information on phylum, class, order, family, genus, and species level for each sample is shown in **Figures 7D–H**. The phylogenetic tree of OTUs at the genus taxonomic level is shown in **Figure 7I**.

**Figure 8:**
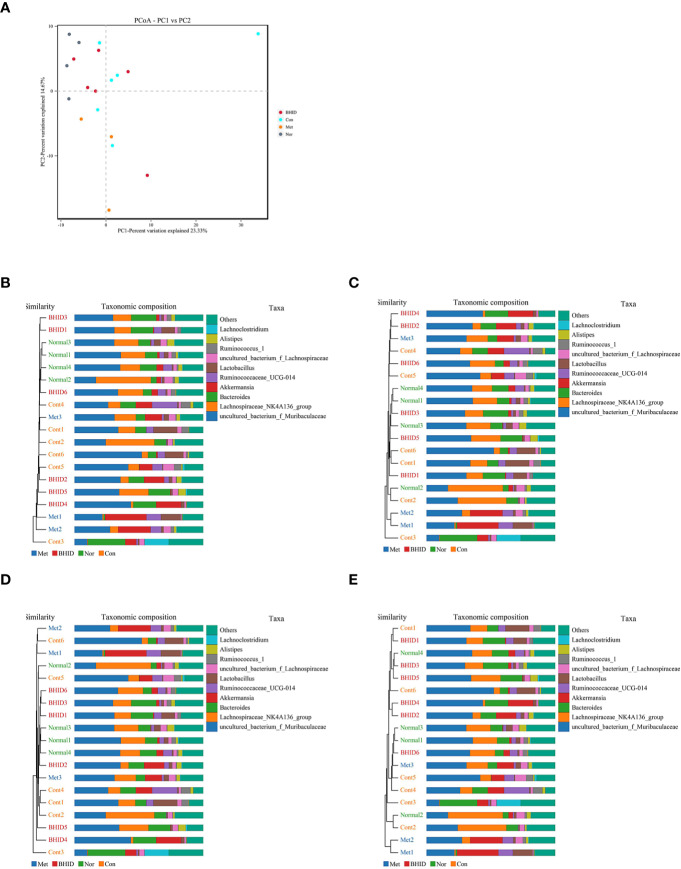
Multivariate analysis for gut microbiota. Weighted UniFrac principal coordinates analysis (PCoA) analysis of gut microbiota based on the operational taxonomic unit (OTU) data **(A)** and Unweighted Pair-group Method with Arithmetic Mean (UPGMA) histogram of cluster tree **(B–E)**, respectively. Nor, normal group; Con, streptozotocin (STZ)–induced diabetic nephropathy (DN) control group; BHID, BHID 500 mg/kg group; and Met, metformin 250 mg/kg group.

To compare the similarity of different samples in species diversity, the principal coordinates analysis (PCoA) (**Figure 8A**) and Unweighted Pair-group Method with Arithmetic Mean (UPGMA) histogram of the cluster tree (**Figures 8B–E**) were obtained by beta diversity analysis. Owing to weighted UniFrac PCoA analysis, the points of the BHID group were widely distributed between the normal and DN control groups, and the metformin groups did not show a significant callback effect. Thus, it is suggested that BHID may be more effective in restoring the structure of gut microbiota towards that observed in the control group compared with metformin.

In our research, Metastats was used to investigate the biomarkers with a statistically significant difference (LDA score>4). There were significant differences between the normal and DN control groups with regard to the flora structures, specifically for: Patescibacteria and Deferribacteres at the phylum level; Saccharimonadia, Actinobacteria, and Deferribacteres at the class level; Saccharimonadales, Corynebacteriales, Deferribacterales, and Enterobacteriales at the order level; Saccharimonadaceae, Atopobiaceae, Rikenellaceae, Corynebacteriaceae Deferribacteraceae, Enterobacteriaceae, and Aerococcaceae at the family level; and Candidatus_Saccharimonas, Peptococcus, f_Erysipelotrichaceae, Anaerovorax, Butyricicoccus, Alistipes, Facklamia, A2, f_Ruminococcaceae, Coriobacteriaceae_UCG-002, Corynebacterium_1, Mucispirillum, Enterobacter, UBA1819, Kerstersia, Acetatifactor, Desulfovibrio, Turicibacter, ASF356, Brevundimonas at the genus level.

After BHID administration, the following significant differences were identified between the control and the BHID treatment groups for the flora structure: Actinobacteria at the phylum level; Coriobacteriia, Oxyphotobacteria, and Erysipelotrichia at the class level; Coriobacteriales, Chloroplast, and Erysipelotrichales at the order level; Atopobiaceae, uncultured_bacterium, Erysipelotrichaceae, Clostridiales_vadinBB60_group at the family level, Peptococcus, Roseburia, Coriobacteriaceae_UCG-002, f_Clostridiales_vadinBB60_group, Kerstersia, and Acetatifactor at the genus level. The above significant differences were further verified by ANOVA (**Supplementary Figure 2**). Correlation analysis was performed and the results are presented as a network diagram (**Supplementary Figure 4**).

### Network Pharmacology Analysis and Verification

To explore the potential ingredients and molecular targets of BHID that have an effect on DN, we conducted a network pharmacological analysis. We identified 153 potentially active compounds and 472 gene targets corresponding to those potentially active compounds in BHID. We also searched for 2,298-related DN target genes. A Venn intersection target diagram was built, and 193 common targets were identified (**Supplementary Figure 4**).

**Figure 9:**
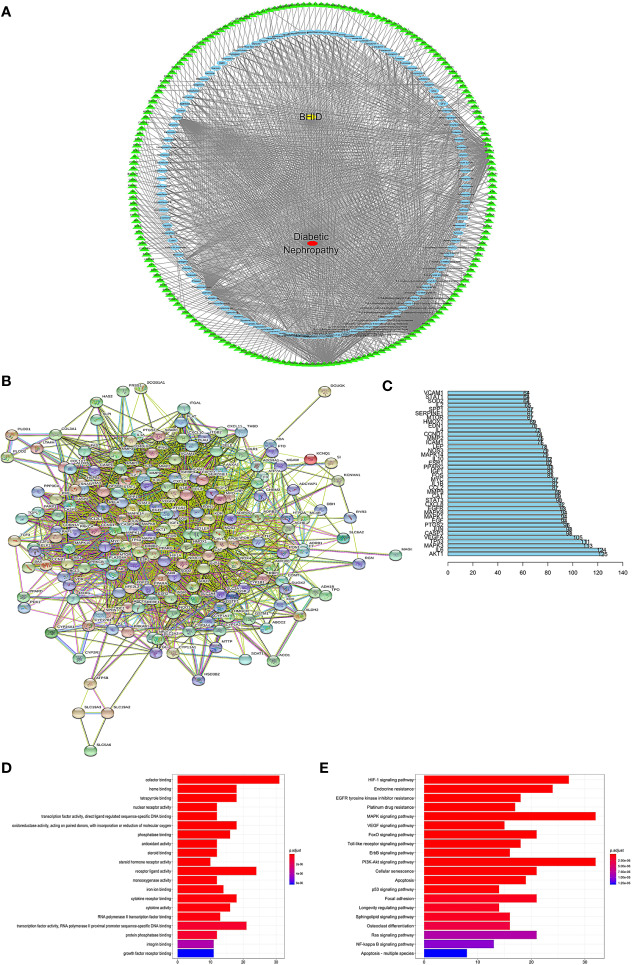
Network construction, pathway, and functional enrichment analysis of the effect of Bekhogainsam decoction (BHID) on diabetic nephropathy (DN). Potential active ingredient-target-disease network **(A)**: Different color symbols as mentioned here: disease (red), BHID (yellow), targets (green), and compounds (blue). Protein-protein interaction (PPI) network **(B)** Node information as mentioned here: query proteins and first shell of interactors (colored nodes), second shell of interactors (white nodes), proteins of unknown 3D structure (empty nodes), some 3D structure is known or predicted (filled nodes), curated databases (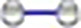), experimentally determined (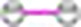), gene neighborhood (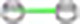), gene fusions (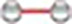), gene co-occurrence (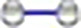), text mining (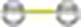), co-expression (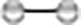), and protein homology (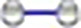). **(C)** Frequency analysis of protein targets. **(D)** Kyoto Encyclopedia of Genes and Genomes (KEGG) pathway enrichment analysis The gradual change in color represented the change in probability. **(E)** Gene Ontology (GO) function analysis. The gradual change in color represents the change in probability.

For a comprehensive identification of the mechanism of BHID on DN, we constructed a network between BHID active components, the corresponding targets, and DN-related genes (**Figure 9A**). In combination with high-frequency node analysis in the PPI network (**Figure 9B**), we were surprised to see AKT1 (protein kinase B1), IL6 (interleukin 6), MAPK3 (mitogen-activated protein kinase 3), and TP53 (tumor protein P53) at the center of the network (**Figure 9C**).

GO (**Figure 9E**) and KEGG (**Figure 9D**) analyses were conducted for pathway enrichment to identify the relevant pathways and functions based on putative targets. Functional analysis revealed that the MAPK signaling pathway and the PI3K/Akt signaling pathway significantly enriched the DN-related pathways. Moreover, functional analysis data revealed that these putative targets not only modulated cell proliferation, apoptosis, growth, and inflammatory response, but also tuned the phosphatase binding and protein serine/threonine kinase (PI3K/Akt) signaling pathways.

**Figure 10:**
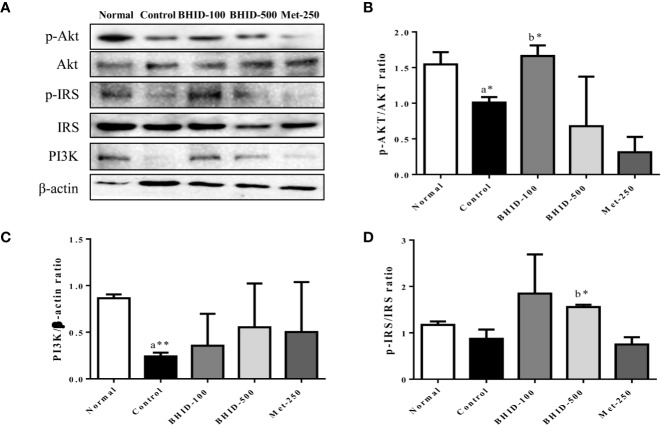
Effects of Bekhogainsam decoction (BHID) on the PI3K/AKT and IRS-1 signaling pathways in the kidney tissue of mice with diabetic nephropathy (DN). Phosphorylation of Akt-Ser473, IRS-Ser307, and PI3K p85 was assessed by western blotting **(A)**. Relative expression of Akt-Ser 473 **(B)**, IRS-Ser307 **(C)**, and PI3K p85 **(D)** was calculated with the total and phosphorylated-forms or β-actin for normalization. Normal, normal group; Control, streptozotocin (STZ)–induced DN control group; BHID-100, BHID 100 mg/kg treatment group; BHID-500, BHID 500 mg/kg treatment group; and Met-250, Metformin 250 mg/kg treatment group. The data are expressed as the mean ± standard deviation (*n*=7). *p<0.05 and **p<0.01 vs. normal (a) or control (b) group.

These data provided a theoretical basis for the possibility that the antagonistic activity of BHID against DN may be related to the activity of PI3K/Akt- and MAPK-related targets. These predicted results were verified by western blotting (**Figure 10**).

Significant inhibition of Akt (*p*<0.05, **Figure 10B**) and IRS-1 (**Figure 10D**) phosphorylation and PI3K (*p*<0.01, **Figure 10C**) expression was observed in the DN control group compared with the normal group. The administration of BHID at low and high doses increased PI3K expression, but the effect was not significant. Moreover, BHID administration significantly increased Akt phosphorylation at low doses (*p*<0.05) and IRS-1 phosphorylation at high doses (*p*<0.05) compared with the control group. Metformin had little effect on the Akt/PI3K and IRS-1 pathways.

**Figure 11:**
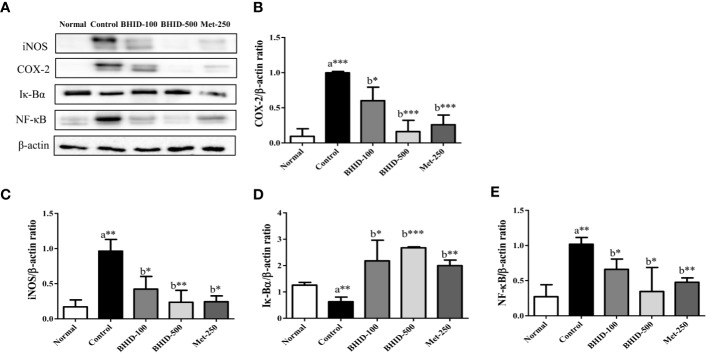
Effects of Bekhogainsam decoction (BHID) on the expression of iNOS, COX-2, I-κB, and NF-κB in the kidney tissue of mice with diabetic nephropathy (DN) **(A)**. The expression of iNOS **(C)**, COX-2 **(B)**, I-κBα **(D)**, and NF-κB p65 **(E)** was assessed by western blotting. The relative expression was calculated with β-actin for normalization. Normal, normal group; Control, streptozotocin (STZ)–induced DN control group; BHID-100, BHID 100 mg/kg treatment group; BHID-500, BHID 500 mg/kg treatment group; and Met-250, Metformin 250 mg/kg treatment group. The data are expressed as the mean ± standard deviation (*n*=7). *P<0.05, **P<0.01, and ***P<0.001, vs. normal (a) or control (b) group.

For the expression of the inflammatory mediators involved in the MAPK pathway (**Figure 11A**), significant increases in iNOS (*p*<0.01, **Figure 11C**) and COX-2 (*p*<0.001, **Figure 11B**) occurred in the DN control group compared with the normal group. However, BHID administration significantly decreased the expression of iNOS (*p*<0.05 for low dose and *p*<0.01 for high dose), and COX-2 (*p*<0.05 for low dose and *p*<0.001 for high dose) relative to the control group. Metformin also significantly inhibited their expression (*p*<0.05 for iNOS and COX-2 for *p*<0.001) in the kidney tissue.

Significant increases in the expression of I-κBα and decreases in the expression of NF-κB were observed in response to BHID at low (*p*<0.05 for I-κBα and *p*<0.05 for NF-κB; **Figures 11D, E**) and high (*p*<0.001 for I-κBα and *p*<0.05 for NF-κB; **Figures 11D, E**) doses compared with the DN control group. Metformin also significantly increased I-κBα expression (*p*<0.01) and decreased NF-κB expression (*p*<0.01) in the kidney tissue.

## Discussion

DN is a diabetic complication known as wasting-thirst disease, Sogal, or Xiaoke syndrome in traditional medicine. BHID is a representative prescription for the control of diabetic symptoms, such as excessive consumption of fluid owing to intense heat in the spleen and stomach, and for the restoration of yin-deficient conditions. One of the constituents of BHID, Gypsum fibrosum is one mineral with hydro calcium fibriform crystallized polymeric and traditionally used to clear heat of lung and stomach at qi system in China and Korea. It has various characteristics, such as heart clearing, wound healing, purging of fire, and promotion of tissue regeneration (Li et al., 2006; Zhang B. et al., 2019). The Anemarrhenae rhizome clears heat and purges fire from the lung and kidney by generating fluids, moistens dryness, and has been reported to exert antithrombotic, antidiabetic, anticolitic, and antiallergic effects in modern pharmacology (Lu et al., 2011; Jang et al., 2013; Li et al., 2013; Makino et al., 2014). Glycyrrhizae radix et rhizoma is the most frequently used crude drug in traditional medicine for the treatment of a variety of diseases (Nose et al., 2017; Wang et al., 2018). Specifically, it contains glycyrrhizin, which is a bioactive compound known to exert antidiabetic activities through the amelioration of insulin resistance, hyperglycemia, dyslipidemia, and oxidative stress (Li et al., 2006; Sen et al., 2011; Sil et al., 2013; Rani et al., 2017). Polished round-grained rice is a rice-grain sprout commonly used to invigorate spleen function, promote digestion, and improve the appetite, and is applied as a source of energy in So-gal syndrome with polydipsia. Ginseng radix et rhizoma is known as the “King of Herbs” because of its varied effects, including improving fitness, preventing fatigue, prolonging life, regulating blood sugar levels, soothing the nerves, and enhancing immunity. Ginseng radix et rhizoma has been shown to exert various pharmacological activities toward specific diseases, such as dementia, diabetes, respiratory infections, and cancer, *via* its constituent compounds, ginsenosides Rb1, Rg1, and Rg3 (Mancuso & Santangelo, 2017; Mohanan et al, 2018). From HPLC analysis, mangiferin and neo-mangiferin in Anemarrhenae rhizoma, liquiritin, and ammonium glycyrrhetate in Glycyrrhizae radix et rhizoma, and ginsenoside-Rg1 in Ginseng radix et rhizoma in BHID were identified as main compounds in BHID. This result will assist in the control the quality of BHID and will support further studies, such as pharmacodynamics experiments.

Experimental studies have tended to focus on the clinical treatment of BHID in DM. The antidiabetic effects of BHID and its components have been known and used for thousands of years to effectively treat DM, and it has been studied in many *in vivo* experiments (Chang et al., 2006). However, little is known about its individual effects on diabetic complications, especially DN, and the mechanisms responsible for these effects. Therefore, we performed a detailed evaluation of the effects of BHID on STZ-induced DN in mice and identified the molecular mechanisms responsible for its effects on DN. STZ is a naturally occurring antineoplastic alkylating agent that is particularly toxic to insulin-producing beta cells in the pancreas and thus induces experimental DM and DN. We induced DN in mice by 3 days’ consecutive STZ injection; conferring systematic and serious histopathological damage in the kidney as well as typical diabetic symptoms, such as hyperglycemia, polydipsia, and polyuria with weight loss. The administration of low and high doses of BHID significantly decreased the increases in food and water intake, urine volume, and blood glucose and triglyceride levels, and significantly increased insulin secretion. These findings indicate that BHID can improve the diabetic symptoms of hyperglycemia, polydipsia, and urorrhagia in DN.

The liver is an important target organ for the regulation of metabolic functions, including insulin sensitivity and gluconeogenesis. The clinical diagnosis of damage to the structural integrity of the liver is usually tested by monitoring the activity of serum AST and ALT. Traditional medicine theory considers the liver and kidney to be homologous; specifically, although their structure and function are different, the origin of the liver and kidney is the same, and their physiological and pathological characteristics are therefore closely related. In the present study, the administration of BHID protected the liver against STZ-induced diabetic toxicity, resulting in decreased AST and ALT levels in DN mice.

The pathogenesis of DN is not fully established; however, it is generally believed to be related to genetics, hyperglycemia, hemodynamic changes, and the production of various cytokines (Piccoli et al., 2015). The glucose transporter (Glut) family is the most important membrane protein for the control of glucose uptake and use, and is distributed widely in the brain, kidney, skeletal muscle, myocardium, and adipose tissues (Colberg et al., 2010). Diabetes with hyperglycemia and insulin deficiency is associated with the loss of GLUT4 expression and translocation, which increases diacylglycerol synthesis (DAG) and activates the PKC pathway (Melloni et al., 1987). The PKC family consists of at least 10 serine/threonine protein kinases that play a central role in various cellular activities, such as the control of growth, differentiation, and apoptosis (Nishizuka, 1986). Therefore, activation of the DAG/PKC signaling pathway can trigger the polyol pathway, oxidative stress, and glycosylated terminal products involved in the occurrence of diabetic complications. This pathway also regulates the permeability of glomerular endothelial cells and, simultaneously, the synthesis and transformation of extracellular matrix in kidney tissue (Sajan and Farese, 2012; Yang and Zhang, 2015). PKC participates in the injury of glomerular podocytes by activation of the TGF-β1 signaling pathway (Xiao et al., 2018). TGF-β1, one of the most critical fibrogenic factors, increases the synthesis of extracellular matrix (ECM) in the mesangial area of the kidney and accelerates thickening of the glomerular basement membrane, thereby inducing glomerular sclerosis and renal failure. Therefore, the PKC/TGF-β1 signaling pathway plays an important role in glomerular sclerosis (Chang et al., 2016) and renal interstitial fibrosis (Wang et al., 2017). Alpha-SMA, a characteristic marker of myofibroblasts, is increased in the kidney tissue during the transformation of renal tubular epithelial cells into mesenchymal cells (Niu et al., 2014). In this study, we found that expression of PKCα, TGF-β1, and α-SMA was increased in the kidney tissue of DN mice, and this increase in expression was markedly suppressed by the administration of BHID. These results indicated that BHID inhibited renal fibrosis in DN through the downregulation of PKCα/TGF-β1/α-SMA expression. We also observed the protective effects of BHID against morphological destruction and fibrosis by using H&E, PAS, and Masson’s trichrome staining.

The theory of “Yin-Yang Balance” and the practice of “BianZheng” in TKM and TCM have many common themes with the holism and systemic theory of systems biology. The concept of systems biology medicine has supported the advancement of TM research for medicine. The study of metabolomics, intestinal flora, and network pharmacology will help to achieve the internationalization and modernization of TM. In our study, BHID altered DN in mice through the inhibition of renal injury, as evidenced by metabolomics coupled with intestinal flora and network pharmacology analyses.

After the anti-DN potential was tested, the mechanism of BHID action was examined by a metabolomics analysis of the serum. The results showed that 36 specific metabolic biomarkers and four main metabolic pathways were affected by BHID treatment. These metabolites and metabolic pathways were closely related to various nutrients, such as carbohydrates, proteins, fat, vitamins, and trace elements (Sarkozy et al., 2014). Moreover, valine, leucine, and isoleucine biosynthesis, which is the most important metabolic pathway for insulin sensitivity (Kaiser et al., 2018), had the highest impact value. Alanine, aspartate, and glutamate metabolism pathways are the signature pathologic metabolic pathways involved the metabolic disorders of DM (Engskog et al., 2017). Moreover, analysis of the gut microbiota verified that these amino acid metabolic disorders may be considered to provide feedback on intestinal flora. Thus, we can confirm the potential and efficiency of BHID in DM or DN through serum metabolomics analysis.

Moreover, BHID can alter microbial composition. The pathogenesis of DM and DN is correlated with inflammation in the intestinal flora. In our study, significant differences were found for Patescibacteria and Deferribacteres at the phylum level between the DN control group and BHID treatment group. More seriously, the worst and serious for the homogeneity and abundance imbalance can be observed in the control group. In combination with the biochemical and pathological indices, we showed that there was an inherent inevitability between the imbalance of intestinal flora and the occurrence of DM or DN. After BHID oral administration, there was a significant difference in Actinobacteria at the phylum level between the control and the BHID treatment group. Actinobacteria is the most important flora that causes intestinal inflammation and ulcers, and stimulates multiple inflammatory reactions (Burge et al., 2019). Our results showed that the administration of BHID can suppress inflammatory reactions by altering the composition of the intestinal flora. More importantly, Coriobacteriia is an important flora for glucose metabolism and the conversion and absorption of medicinal ingredients that can improve the symptoms of DN or STZ-induced DN in mice (Kikuchi et al., 2019). Moreover, the oral administration of BHID can improve the growth of probiotics, such as Clostridiales and Peptococcus, to maintain the homeostasis of the intestinal flora. Recent research has shown that the effects of Ginseng radix et rhizoma and Anemarrhenae rhizoma on obesity, cancer, and DM are mediated through the regulation of the composition of the intestinal flora (Xiao et al., 2016; Dong et al., 2019). In our study, significant differences were observed in the structure of the intestinal flora at the phylum, class, order, family, genus, and species levels between the normal and control, and control and BHID-treatment groups, and a high frequency of interconnected flora was also summarized. To the best of our knowledge, the present study is the first to demonstrate the effect of BHID or its components on the modulation of gut microbiota.

To further understand the molecular mechanism of the effects of BHID on DN, a network between BHID active components, the corresponding targets, and DN-related genes was established based on network pharmacology analysis. We concluded that BHID antagonism against DN may be related to the activity of PI3K/Akt- and MAPK-related targets. This hypothesis was further verified by western blotting. Beta-cell apoptosis in the pancreas, insulin resistance, and hyperglycemia induce the excessive production of inflammatory mediators, iNOS, and COX-2, through activation of the NF-κB signaling pathway in DN (Lu et al., 2014). Moreover, the phosphorylation of serine residues in the insulin receptor (IRS-1) results in diminished enzymatic activity in the PI3K/Akt pathway in DN. Previous studies have reported that the NF-κB pathway was activated in various tissues, including kidney tissues of DN (Wang et al., 2015; Gao et al., 2018). The PI3K/Akt pathway is one of the TGF-β1 downstream signaling pathways that can resist podocyte apoptosis by controlling purine adenosine, protein overload, hemodynamic disorder, and other conditions, and can give rise to proteinuria in DN (Saleem, 2015). In this study, we found that BHID administration in mice with STZ-induced DN effectively inhibited the expression of iNOS and COX-2 in the kidney tissue of mice with through the suppression of the I-κB/NF-κB signaling pathway, as well as activation of the PI3K/Akt signaling pathway.

Moreover, metformin is the most common classical drug used in the clinical treatment of T2DM; it can increase glucose utilization in the body by peripheral cells, prevent liver and kidney glycogenogenesis, reduce the synthesis and output of glucose by the liver and kidney, and inhibit glucose uptake by cells in the intestinal wall (Hostalek et al., 2015). It suppresses glucose synthesis from multiple links, promotes glucose conversion and absorption, and reduces blood glucose levels in patients with diabetes (van Stee et al., 2018). However, long-term use causes nausea, vomiting, diarrhea, fatigue, and other adverse reactions (Hostalek et al., 2015). In our results, BHID effectively decreased the kidney-to-body ratio, urine volume, serum levels of glucose, AST, and ALT, and increased insulin- and HDL-cholesterol compared with metformin. With regard to kidney damage, BHID reduced fibrosis, inflammation, and insulin resistance through the inhibition of TGF-β1 and COX-2 expression, and the activation of Akt and IRS-1 compared with metformin. These results indicated that BHID is a good candidate for traditional medicine prescription and as an alternative to metformin for the treatment of DN in clinics.

To the best of our knowledge, our study is the first to report the anti-DN effects of BHID in a mouse model, as shown by metabolomics coupled with analysis of the intestinal flora and network pharmacology, in which the holistic view of systems biology runs through the whole study. However, the content of the five main chemical substances in the BHID was measured, and the possible changes in the chemical compositions of each drug during the decocting process and metabolic labeling *in vivo*. In our study, we aimed to establish the internal relevance between metabolomics, intestinal flora, and network pharmacology with limited evidence; therefore, further explanation and evidence should be identified and explored to strengthen this relevance, and the core metabolites should be determine.

## Conclusion

The administration of 100 and 500 mg/kg BHID in mice with STZ-induced DN for 4 weeks improved diabetic conditions, such as physiological and serological imbalances and damage to the liver and kidney. In the analysis of metabolomics coupled with gut microbiota and network pharmacology, BHID was shown to alter the metabolites and gut floral structures, and the protective effect of BHID against the DN-induced damage to the kidney is underlying the activation of the PI3K/MAPK pathway. Our findings suggested that there is scientific evidence for the beneficial effects of BHID in the clinical treatment of patients with diabetes and for the prevention of DN progression.

## Data Availability Statement

The raw data supporting the conclusions of this article will be made available by the authors, without undue reservation, to any qualified researcher.

## Ethics Statement

All animals were handled according to the animal welfare guidelines issued by the Korean National Institute of Health and the Korean Academy of Medical Sciences for the care and use of laboratory animals and approved by the Institutional Animal Care and Use Committee of Dongguk University (IACUC-2017-012).

## Author Contributions

Y-KP and HJ designed the study. XM, JM, SK, AK, and HJ performed the experiment and conducted a statistical analysis. XM and HJ wrote the manuscript. All authors revised the manuscript and approved the final version of the manuscript.

## Funding

This research was supported by the Basic Science Research Program through the National Research Foundation of Korea (NRF) funded by the Ministry of Education (Grant No. 2016R1D1A2B01012117 & 2016R1D1A1B04935601).

## Conflict of Interest

The authors declare that the research was conducted in the absence of any commercial or financial relationships that could be construed as a potential conflict of interest.

## Footnotes

1 http://www.hmdb.ca/

2 http://www.genome.jp/kegg/

3 http://www.metaboanalyst.ca/faces/home.xhtml

4 http://matplotlib.org/index.html

5 http://lsp.nwu.edu.cn/tcmsp.php/

6 http://bionet.ncpsb.org/batman-tcm/

7 http://www.genecards.org/

8 http://www.omim.org/

9 https://cytoscape.org/

10 https://string-db.org/cgi/input.pl/

11 https://david.ncifcrf.gov/
