# Supplementary material for: A Novel Approach Based on Metabolomics Coupled With Intestinal Flora Analysis and Network Pharmacology to Explain the Mechanisms of Action of Bekhogainsam Decoction in the Improvement of Symptoms of Streptozotocin-Induced Diabetic Nephropathy in Mice
Source: Front Pharmacol. 2020 May 21;11:633. doi: 10.3389/fphar.2020.00633 (PMC7253635; doi:10.3389/fphar.2020.00633)
Supplement: Supplementary file 1 [file DataSheet_1.doc]

Supplementary Material


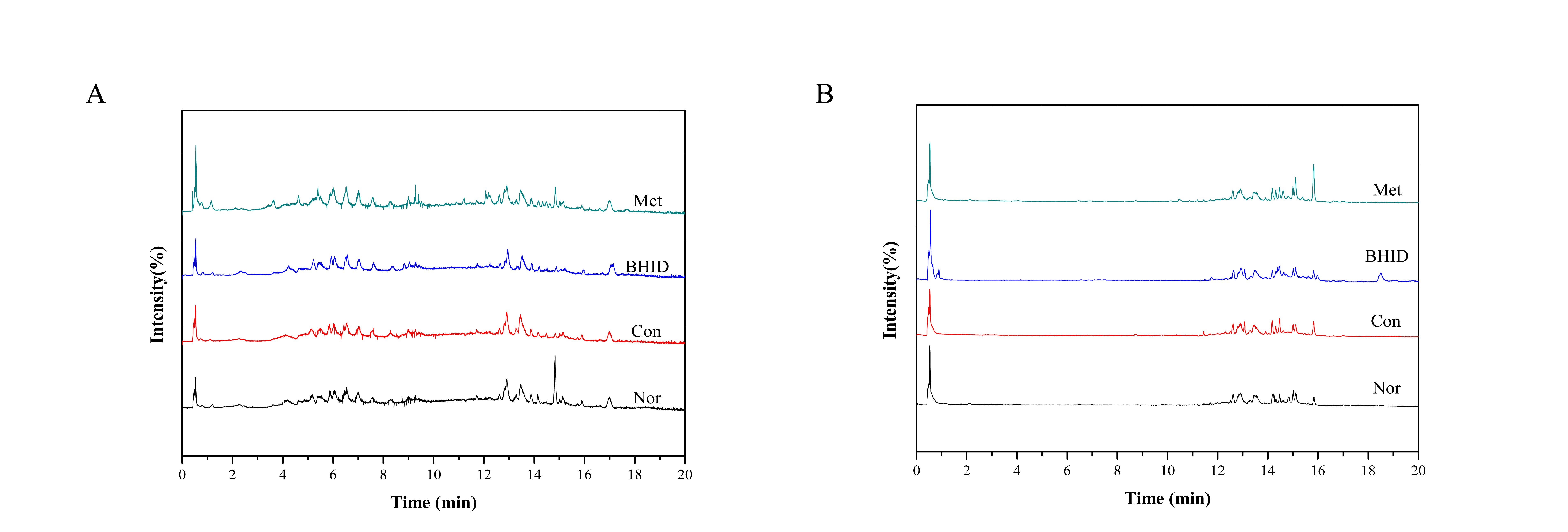


**Supplementary Figure 1.** Total ion chromatograms of mice serum in the positive (A) and negative (B) ion modes.


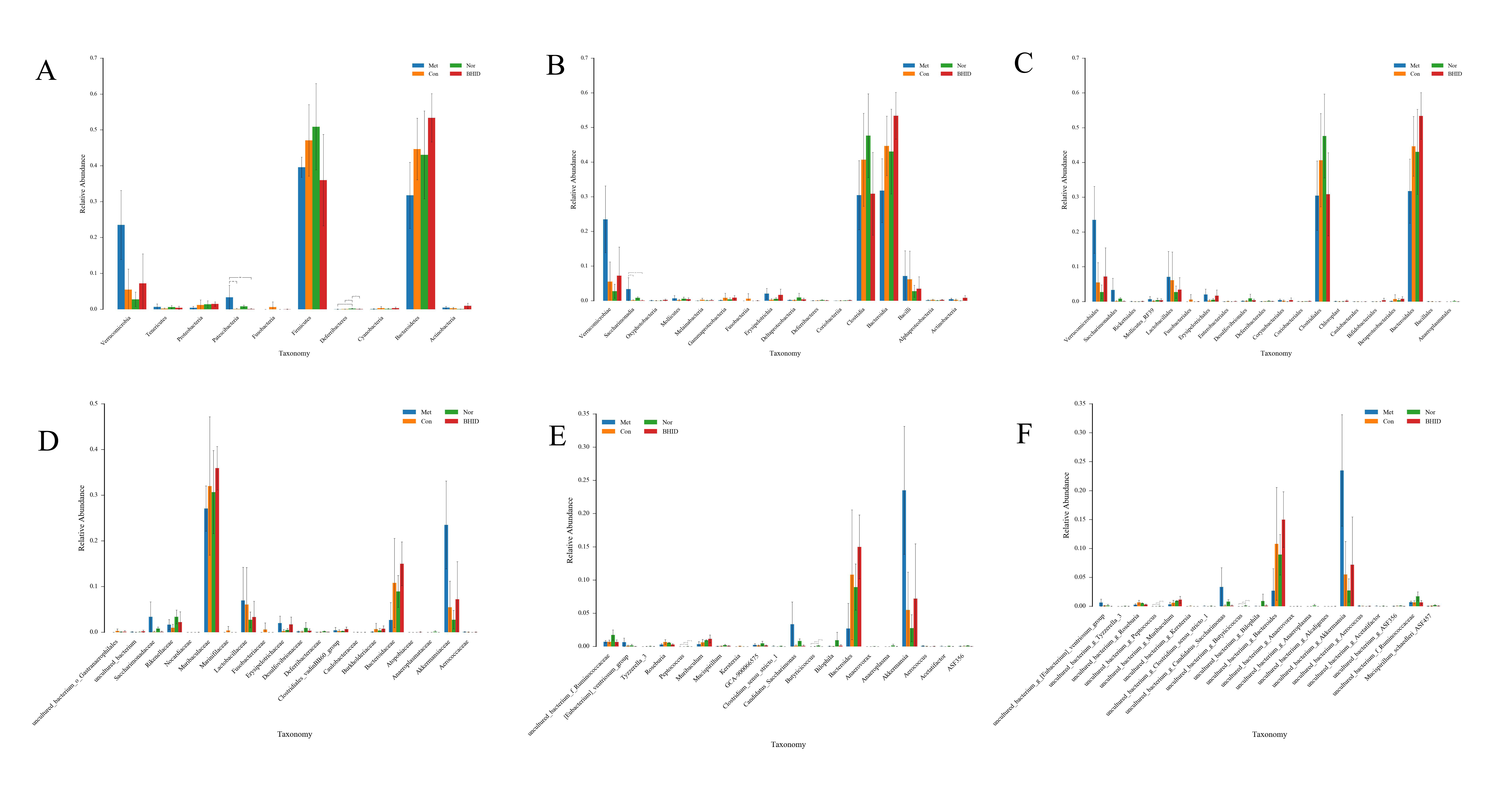


**Supplementary Figure 2.** ANOVA analysis for gut microbiota.

(a, b, c, d, e and f) ANOVA analysis on phylum, class, order, family, genus, species level for each group, respectively.


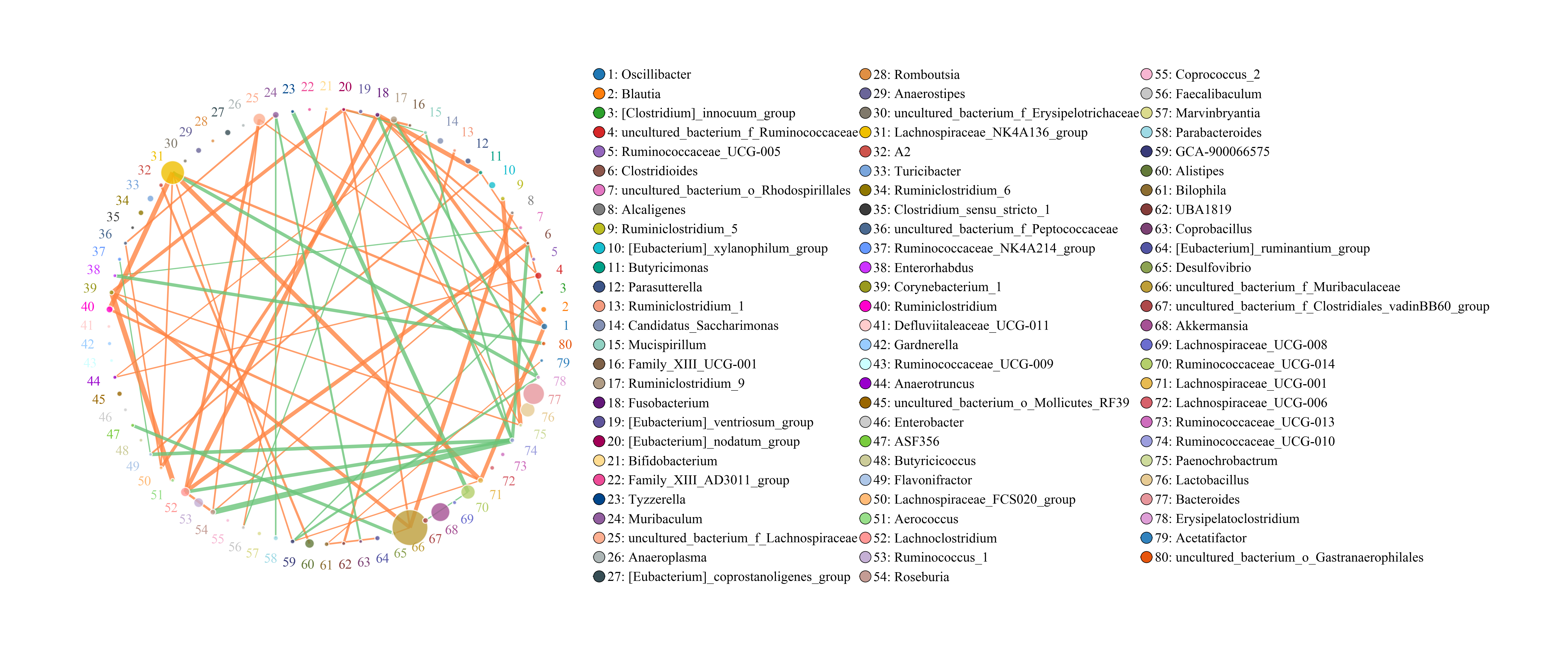


**Supplementary Figure 3.** Correlation analysis for gut microbiota.

The size of a node indicates the frequency of connections. Lachnospiraceae_NK4A136_group, f_Muribaculaceae, Akkermansia, Bacteroides, Lactobacillus, Ruminococcaceae_UCG-014 and f_Lachnospiraceae were high frequency of interconnected floras.


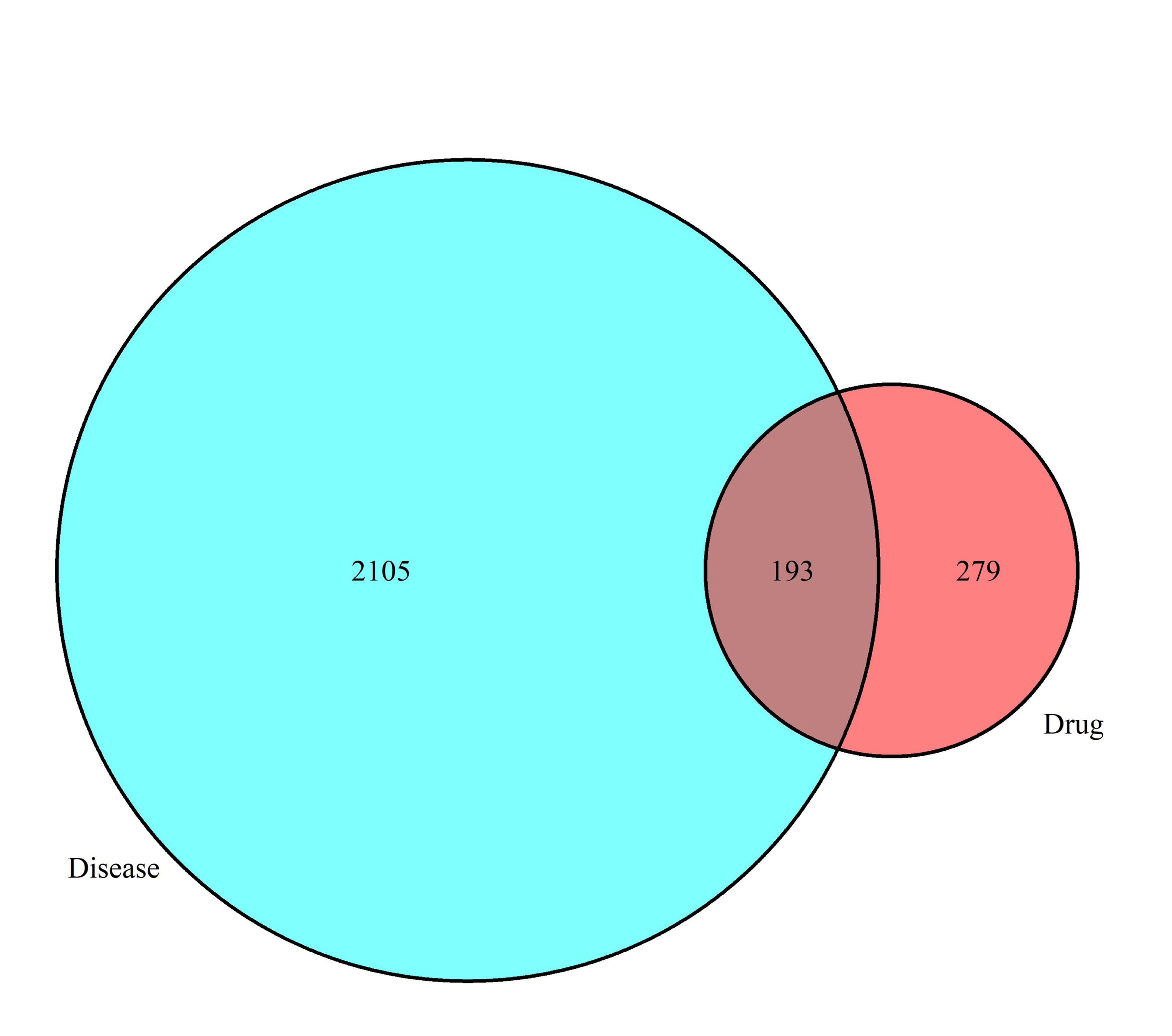


**Supplementary Figure 4.** Venn intersection targets diagram.
